# Problem-Elephant Translocation: Translocating the Problem and the Elephant?

**DOI:** 10.1371/journal.pone.0050917

**Published:** 2012-12-07

**Authors:** Prithiviraj Fernando, Peter Leimgruber, Tharaka Prasad, Jennifer Pastorini

**Affiliations:** 1 Centre for Conservation and Research, Rajagiriya, Sri Lanka; 2 Smithsonian Conservation Biology Institute, Front Royal, Virginia, United States of America; 3 Department of Wildlife Conservation, Battaramulla, Sri Lanka; 4 Anthropologisches Institut, Universität Zürich, Zürich, Switzerland; Australian Wildlife Conservancy, Australia

## Abstract

Human-elephant conflict (HEC) threatens the survival of endangered Asian elephants (*Elephas maximus*). Translocating “problem-elephants” is an important HEC mitigation and elephant conservation strategy across elephant range, with hundreds translocated annually. In the first comprehensive assessment of elephant translocation, we monitored 16 translocations in Sri Lanka with GPS collars. All translocated elephants were released into national parks. Two were killed within the parks where they were released, while all the others left those parks. Translocated elephants showed variable responses: “homers” returned to the capture site, “wanderers” ranged widely, and “settlers” established home ranges in new areas soon after release. Translocation caused wider propagation and intensification of HEC, and increased elephant mortality. We conclude that translocation defeats both HEC mitigation and elephant conservation goals.

## Introduction

Translocation is defined as the ‘deliberate and mediated movement of wild individuals or populations from one part of their range to another’ [1]. It is a commonly used tool in conservation, for establishing, re-establishing and augmenting populations of managed species [2]. It is also employed in managing ‘problem-wildlife’, although a number of studies have questioned its use in this context [3]–[6]. Due to ethical concerns and mounting objections to lethal control [3], [7], translocation is increasingly viewed as a panacea for all wildlife problems [6]. The main objective of problem-animal translocation is eliminating problems caused by wildlife [6] and secondly, saving the animals responsible. In conservation use, translocated animals are usually released in ‘empty’ habitats [1]. In problem-animal translocation they are more likely released in areas fully occupied by conspecifics [3]. Translocated animals may be first acclimatized at the release site (soft-release) or released immediately (hard-release), the latter being more common in problem-animal translocation [2], [3]. Many thousands of problem-animals are translocated annually [3], [4]. Mostly applied to ‘nuisance’ or ‘dangerous’ animals it is taxonomically biased towards mammals. Species so translocated include squirrels [8], raccoons [9], deer, bear, rodents [10], wolves [11], foxes, wild cats [12], cougars [13], leopards [14], tigers [15], elephants [16], [17], geese [18], eagles [19], Gila monsters [20], snakes [21] and crocodilians [22].

The Asian elephant (*Elephas maximus*) is an ‘endangered species’ on the IUCN Red List and is listed in CITES Schedule I [23], [24]. The global population estimate for Asian elephants is 35,000–50,000 [24], [25], one tenth that of African elephants (*Loxodonta africana* and *L. cyclotis*). Asian elephants are now extinct in 78% of their historic range [26]. Currently they are limited to a number of fragmented and isolated populations in 13 south and south-east Asian states [24], [25], [27]. With only 16% of their remaining range protected [27], most Asian elephants are compelled to share space with humans, leading to frequent conflict. For example, over 70% of about 6,000 elephants in Sri Lanka live outside protected areas, where annually human-elephant conflict (HEC) claims the lives of over 70 humans and 200 elephants [28]. Today, HEC is a major conservation, socio-economic and political issue across Asian elephant range [25].

Elephant social organization is sexually dimorphic with group-living adult females and young, and mainly solitary adult males [29]–[31]. Males display a higher propensity for crop raiding, accessing superior resources to gain in size hence reproductive advantage, in a ‘high-risk high-gain’ strategy [32]. Some males raid crops, break into houses for stored grain, and react aggressively to confrontation, causing human injury and death. Considered ‘problem-elephants’, such individuals are responsible for the majority of HEC incidents [33].

While lethal control is preferred in some parts of Africa [34], translocation remains one of the main elephant management tools and hundreds of elephants are translocated annually [17], [28], [35], [36]. Translocating problem-elephants aims to mitigate HEC by removing them from human proximity. It also attempts to further elephant conservation, assuming higher mortality if problem-elephants remain in their original home ranges. The *modus operandi* for translocating problem-elephants is capture by drug immobilization, transport by truck and release in a protected area. In Sri Lanka and India, elephants so translocated are exclusively males, while in Malaysia, Indonesia and some African countries it may involve both sexes [35], [37].

Elephants have comparatively large home ranges and can cover long distances quickly [38]–[40]. Often they also inhabit poor visibility habitat and actively avoid humans [39], [41]. Consequently, monitoring individual elephants without radio-telemetry is ineffective and with VHF transmitters is at best difficult. Only a few translocations have been previously monitored with radio-telemetry, consisting of one elephant in India [40], 11 in Kenya [35] and six in South Africa [42] that were tracked with VHF, two tracked with satellite-PTT transmitters in Malaysia [37] and one with GPS in Kenya [35]. Anecdotal accounts [43]–[45] and the few monitoring studies, suggest that some translocated elephants return while others settle in release areas.

In this paper, we report on the first comprehensive assessment of problem-elephant translocation. Using remote-download GPS collars, we monitored 12 males translocated 16 times and 12 males resident in their normal home ranges. Here we compare and contrast the behavior and HEC involvement of translocated and resident elephants, and discuss the relevance of findings for management.

## Methods

### Study Animals

All elephants in our study were adults and were classified as ‘mature-adults’ or ‘young-adults’, corresponding approximately to above and below 30 years of age. Individuals displaying a combination of the following characters were identified as ‘mature adults’: shoulder height over 270 cm; well developed secondary sexual characters such as wide trunk base, prominent nasal protuberance, deep temporal depression and large penis/penile bulge; characters indicating active musth such as temporal gland discharge and urine dribbling; and age related characters such as completely folded top edge of ear and heavy de-pigmentation [46], [47].

All 12 translocated elephants were identified as ‘problem-elephants’ by the Department of Wildlife Conservation Sri Lanka (DWC) based on HEC incidents and information from villagers. The resident males consisted of two (Kandula and Kavan) that did not cause HEC and 10 problem-elephants. Reported incidents of crop raiding, house breaking or human injury and death, and entering areas of human habitation by monitored elephants were taken to indicate causation of HEC.

### Collars and Collaring

Translocated elephants were fitted with radio-collars at the time of capture. The collars consisted of a GPS unit, VHF transmitter beacon, satellite or GSM transmitter for data download (Table 1) and batteries packaged into one integrated unit. Sky orientation of the functional unit for satellite detection was achieved by a counterweight. Collars that became non-functional were not removed as it was determined that the risk to the elephant and collaring team in tranquilization was not acceptable for the purpose of collar removal. Collar belting degraded and broke off within a period of 2–4 years (unpublished data).

**Table 1:** Details of collars, programming and use-area (MCP) for the translocated elephants.

Category	Animal ID	Collar Make	Model	DataTrans-mission	GPSInterval[hours]	TrackingPeriod[days]	MCP [km^2^]
Homers	Chandi	Telonics	Gen. IV	Argos	8	116	4,380.4
	Homey	Telonics	Gen. III	Argos	4	217	531.3
	Homey	Telonics	Gen. III	Argos	4	284	846.7
	Homey	Telonics	Gen. III	Argos	4	17	435.1
	Kabaraya	Africa Wildlife Tracking	SEL-201	Satellite	8	92	571.4
Wanderers	Babar	Telonics	Gen. IV	Argos	4	35	1,373.2
	Brigadier	Vectronic	2007	SMS	4	178	2,067.1
	Ravana	Telonics	Gen. III	Argos	4	91	527.6
	Ravana	Telonics	Gen. III	Argos	4	244	163.9
	Siyak	Vectronic	2007	SMS	4	99	1,274.0
	Wasaba	Telonics	Gen. III	Argos	4	585	3,669.6
Settlers	Ekes	Telonics	Gen. IV	Argos	8	1009	162.1
	Galli	Telonics	Gen. III	Argos	4	739	1,026.0
	Nalagiri	Telonics	Gen. IV	Argos	8	160	138.4
	Tzu Chi	Africa Wildlife Tracking	SEL-201	Satellite	8	279	205.5
	Tzu Chi	Africa Wildlife Tracking	SEL-201	Satellite	8	55	60.4

### Translocation

**Figure 1:**
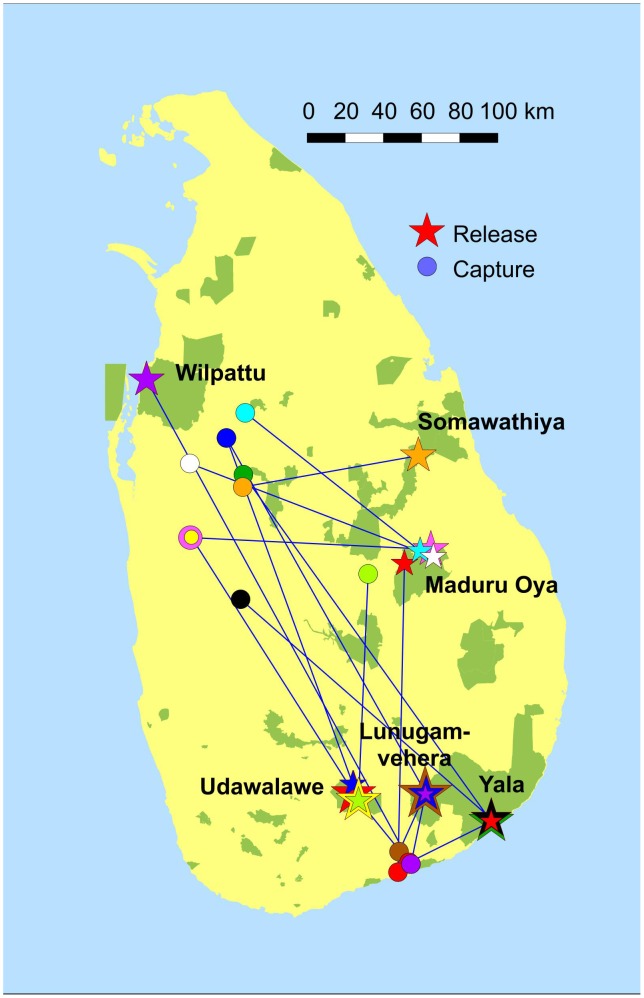
Map of translocations. Circles indicate capture sites and stars release sites. Different colors denote individual elephants. Green polygons represent protected areas under the Department of Wildlife Conservation.

All translocated elephants were captured outside protected areas and released inside national parks (Fig. 1). All release locations were within current elephant range and had ample water and fodder. Two males (Ravana and Tzu Chi) were translocated twice and one (Homey) was translocated three times. Translocated elephants were ‘hard-released’ and the time from capture to release was 1–3 days.

Translocated elephants were tracked using the VHF beacon on the collar and observed opportunistically.

### Ethics Statement

The study was mandated by the DWC and conducted collaboratively by the DWC and the Centre for Conservation and Research (CCR). Under the ‘Fauna and Flora Protection Ordinance’ of Sri Lanka, the DWC is legislated as the government institution that is the sole authority on wildlife management in Sri Lanka and there is no requirement or procedure to obtain separate approval for activities conducted by the DWC. Elephants were captured and translocated as part of the routine activities of the DWC for mitigating HEC and conserving elephants. Collaring of resident elephants was done as part of another on-going study by the DWC and CCR to obtain baseline information to better elephant conservation and HEC mitigation. Tranquilizing elephants for collaring was done by a DWC team of 15–20 personnel led by two DWC veterinarians according to guidelines set out by the DWC.

All efforts were made to prevent and minimize suffering of animals concerned and to ensure the safety of animals and personnel involved in research activities. Radio darts were used to maximize the safety of darted animals by reducing search time and minimizing possibilities of complications of tranquilization under field conditions. Throughout the tranquilized period, a veterinarian monitored the status of the elephant to prevent any complications. Tranquilized elephants were given a health check and were treated by wound cleaning and injection of antibiotics as indicated (eg. gunshot wounds, abscesses).

### Data Analysis

Collars were programmed to collect GPS locations every 4 or 8 hours and transmit the data every 8, 24 or 48 hours (Table 1). In Telonics and Vectronic collars data were also stored on-board and were directly downloadable if the collar was recovered.

Data received from collars were processed with the corresponding manufacturer’s software. GPS locations obtained were tabulated in Excel, exported into ArcMap (EsriArcGIS) version 9.2 or Quantum GIS version 1.7 (QGIS) and plotted on satellite imagery or 1∶50,000 topographic sheets. Home ranges and ‘use areas’ were calculated as 100% Minimum Convex Polygons in QGIS (single minimum convex hull function).

To simplify directional analysis we re-projected the movement data after release so that all elephant release sites were at the coordinate origin (0,0) and capture sites were oriented at 180° (to the left) from the release location. To assess movement orientation after release, we calculated the spatial mean of all GPS positions acquired during the first 10 days of tracking and computed the movement angle between the release site and this spatial mean. Angles <90° and >270° (in right hemisphere) were taken to represent movement orienting away from the capture site, and all others (90°–270°) towards the capture site. To test whether elephants more often oriented towards the capture site than expected by chance alone, we used a binomial test and calculated confidence intervals. All data manipulations and statistical tests for assessing movement direction were performed using R statistical software (R Development Core Team 2011, <www.R-project.org>).

## Results

Translocated and resident individuals were tracked for periods of 262.5±279.4 (range 17–1,009) and 314.8±298.6 (range 34–1,022) days respectively, giving total periods of 4,200 days of translocated and 3,777 days of resident elephant tracking (Tables 1 and 2). The mean translocation distance was 134.8±72.7 (range 37.4–289.1) km (Table 1). All translocated elephants were released inside national parks. Two elephants were shot dead within the parks where they were released (Tzu Chi and Ravana) and all the others left those parks (time to exit: 33.3±69.3, range 1–263 days, Table 3).

**Table 2:** Details of collar programming and home ranges (MCP) for resident elephants.

Animal ID	GPS Interval[hours]	Tracking Period [days]	MCP[km^2^]
Bandara	4	45	77.4
Kandula	4	1022	98.0
Karattaya	4	270	113.4
Kavan	4	307	62.8
Mahasen	4	41	263.0
Parakum	4	34	196.6
Thaga	4	196	169.6
Wira	4	751	630.5
Dase	1	302	642.9
Hura	1	365	363.8
LokuMaama	1	105	170.8
Tharaka	1	339	594.2

**Figure 2:**
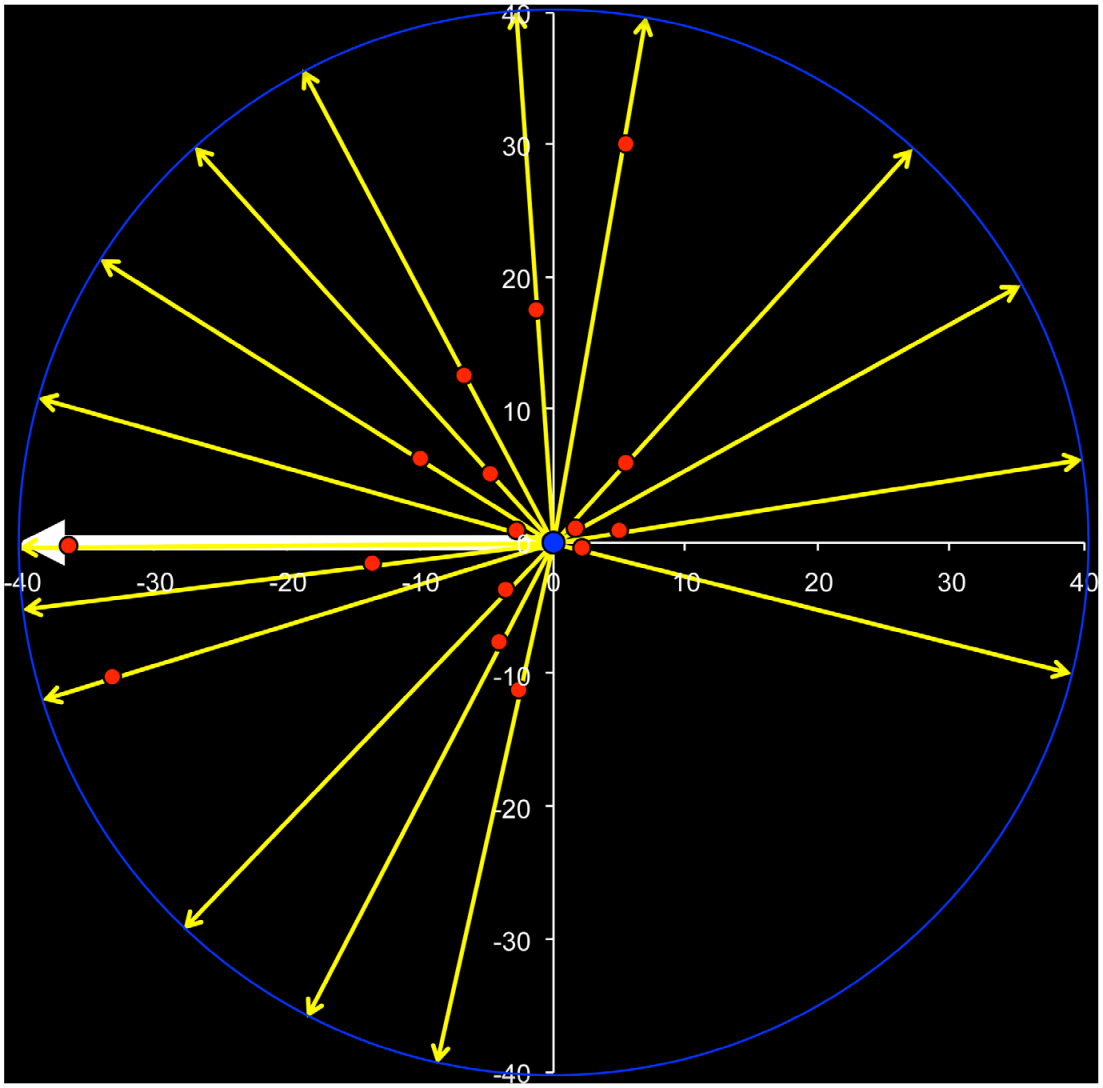
Post-release orientation (yellow arrows) of translocated elephants relative to capture site (white arrow). The blue circle denotes the release point for all elephants and red circles the spatial means of GPS locations over the first 10 days post-release for individual elephants. The binomial probability of the number of elephants orienting towards the capture location (left hemisphere) vs away (right hemisphere) was 0.69 (n = 16, p = 0.11, 95% CI 0.44; 0.86).

Over the first 10 days post-release, in 11 of 16 translocations, elephants oriented towards the capture site (Fig. 2). No aggression was observed between translocated elephants and resident park elephants, and no injuries caused by other elephants were observed on the five translocated males that died (Table 3). All areas where translocated elephants settled had resident elephants. Two elephants (Galli and Ekes) were observed to associate with resident bulls post-release.

**Table 3:** Data summary for translocated elephants.

			Release			Outcome			
Category	Animal ID	Age	Distance [km]	Date	National Park	First day outside park	CausedHEC	Raided crops	Elephant killed
Homers	Chandi	Mature Adult	93.4	15.02.2009	Somawathiya	6	yes	yes	
	Homey	Mature Adult	48.2	19.03.2006	Yala	3*	yes	yes	
	Homey	Mature Adult	46.2	23.10.2006	Udawalawe	39*	yes	yes	
	Homey	Mature Adult	161.7	5.08.2007	MaduruOya	3	yes	yes	yes
	Kabaraya	Mature Adult	116.8	15.09.2010	MaduruOya	8*	yes	?	
Wanderers	Babar	Young Adult	223.4	22.03.2010	Yala	19	?	?	
	Brigadier	Young Adult	126.2	29.04.2010	MaduruOya	1	yes	yes	yes
	Ravana	Young Adult	193.2	20.09.2007	Udawalawe	3	yes	yes	
	Ravana	Young Adult	193.9	20.12.2007	Lunugamvehera	(died inside)	yes	yes	yes
	Siyak	Mature Adult	163.5	19.07.2007	Udawalawe	1*	yes	?	
	Wasaba	Young Adult	118.9	1.07.2006	Udawalawe	13*	yes	no	
Settlers	Ekes	Mature Adult	33.2	12.01.2009	Lunugamvehera^#^	2*	yes	yes	
	Galli	Young Adult	174.6	11.09.2007	Yala^#^	263*	yes	no	
	Nalagiri	Young Adult	136.7	29.06.2009	MaduruOya	76	yes	yes	yes
	Tzu Chi	Young Adult	37.4	15.09.2009	Lunugamvehera^#^	29*	yes	yes	
	Tzu Chi	Young Adult	289.1	23.06.2010	Wilpattu	(died inside)	yes	yes	yes

* Elephant broke through electric fence on a park boundary.

#^‘^Holding ground’, which is a specially fenced off portion (25.5 km^2^) of the park.

? No data.

### Individual Variation in Response

We classified the translocated elephants as ‘homers’, ‘wanderers’ and ‘settlers’ based on response.

**Figure 3:**
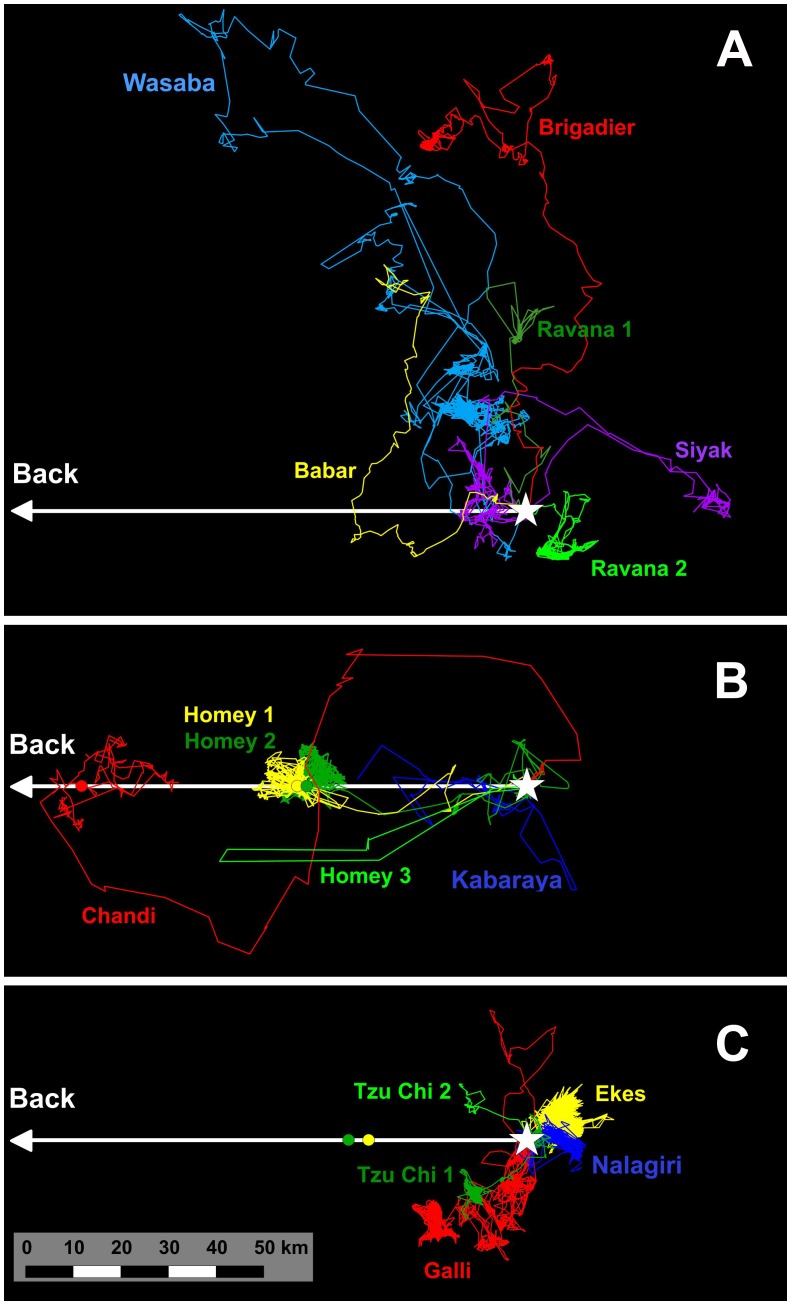
Ranging patterns of translocated elephants. Colored lines show movement tracks of individual elephants re-projected with the release location at 0.0 on an x–y axis and the capture location oriented to the left (white arrow). Color coded circles denote capture locations for translocation distances <100 km. **A** = Wanderers, **B** = Homers, **C** = Settlers.

In five translocations ‘homers’ Chandi, Homey and Kabaraya returned to the capture site thrice and showed movements consistent with successful homing twice (Fig. 3B). Chandi translocated 93.4 km, returned in 29 days. Homey after his first and second translocations over 48.2 and 46.2 km homed back in 5 and 41 days respectively. Homey on his third translocation of 161.7 km showed homing movement for 62.0 km in 4 days but entered a town causing conflict. Chased back to the release location, he settled at the perimeter of the park, raided surrounding villages, was shot repeatedly and died 15 months after from gunshot injuries. Kabaraya translocated 116.8 km, after an initial period in the release area, showed homing movement. However, the collar stopped functioning at 92 days, 81.4 km from the capture point. Homey and Kabaraya showed well directed homing movements while Chandi took a more circuitous route back (Fig. 3B).

‘Wanderers’ Wasaba, Siyak, Brigadier, Ravana, and Barbar showed misdirected long distance movements (Fig. 3A). Wasaba and Siyak travelled 127.0 and 43.0 km respectively till obstructed by the sea, returned and settled proximal to the release area. Brigadier showed directional movement for 95.9 km. When confronted by the sea he swam out, was providentially spotted 5 km offshore by the Sri Lanka Navy, noosed underwater by scuba divers and brought back to shore. He then settled in a new area, continued to cause conflict and died from falling into a well 6 months after. Ravana entered a major town, created conflict and was shot in the leg. He then took refuge in a forest patch where he remained for 3 months. He was recaptured and translocated to another national park, raided cultivations within the park and was found shot dead 8 months later. Babar traveled 95.9 km in 19 days before exiting the park where he was released and the collar came off 16 days later.

‘Settlers’ Galli, Ekes, Tzu Chi and Nalagiri settled proximal to the park where they were released, without any directional long distance movements away from the release site (Fig. 3C). Galli shifted his ‘new’ home range twice after 6 and then 3 months. Galli’s first home range was in the park (176 km^2^) and the others (115 and 73 km^2^) outside. Between his first and second home ranges, for 2 months Galli used only an 8 km^2^ area along a perimeter electric fence of the park. Ekes’ new home range was 162 km^2^, largely adjacent to the park where he was released. He ventured back into the park 16 times, spending 35 days within, in the 1,009 days period tracked. He raided regularly, making nocturnal forays into villages and taking cover in forested habitat during day. Tzu Chi was translocated 37.4 km northeast from his capture site. After 29 days he left the park and two weeks later settled in an area 8.1 km south of the release point, where he continued to cause conflict. He was re-captured eight months later and translocated 289.1 km northwest from the original capture site. Upon release he moved south and was found shot dead 55 days later, 18.3 km from the release point and 355 m from the park boundary. Nalagiri established a new home range partly outside the park where he was released, regularly raided nearby villages and was found shot dead 5 months after release.

### Relation to Conflict

On six instances translocated elephants confronted electric fences on park boundaries. Another two elephants were released within a ‘holding ground’ with a high specification perimeter electric fence plus ‘elephant-trench’ (Table 3). None of them were contained by such barriers, except Ravana who was killed within the park. Translocated elephants had average ‘use-areas’ of 1,090±1,276 km^2^, (range 60–4,380 km^2^; Table 1). The 12 resident males had significantly smaller home ranges of 282±222 km^2^ (range: 63–643 km^2^; Mann-Whitney U test, *P* = 0.0488, Table 2). Homey had home ranges of 153 km^2^ and 132 km^2^ between his translocations and ‘use-areas’ of 311, 570 and 435 km^2^ during them. Chandi had a ‘use-area’ of 4,126 km^2^ during the translocation and a home range of 336 km^2^ after return.

Four of the 12 translocated elephants (Homey, Chandi, Wasaba, Ravana) but none of the 12 resident males entered major towns. The incursions created chaos with human injury and death, damage to property including vehicles and killing of a water-buffalo. The 12 translocated elephants killed 5 people. No deaths were caused by the 12 resident males, one of whom was shot dead during the study period.

## Discussion

### Post-Release Response

The majority of translocated elephants displayed post-release movements oriented towards the capture site (Fig. 2). Homing upon translocation has been observed in a range of species, including bears [48], [49], cougars [13], wolves [11], foxes [12], deer [50], elephant seals [51], eagles [19], crocodiles [22], Gila monsters [20], and newts [52]. Home ranges and spatial organization of individuals reflect resource use and strategies adopted by individuals to maximize fitness [53], [54]. Familiarity with one’s environment and neighbors is positively correlated with individual fitness [55]. Thus, the drive of translocated animals to return, maybe due to the increased fitness accruing from occupying a familiar home range. In long-lived and highly social species such as elephants, selection on home range fidelity, hence drive to home back is likely to be stronger.

Asian elephants have well defined home ranges with high fidelity [39], [56] and it is likely that translocated elephants left the parks where they were released, in attempts to return ‘home’. All six parks where elephants were released had abundant water, wild fodder and female herds. Thus, it is unlikely that the decision to leave was related to resource deficiency. Some translocated elephants associated with resident park elephants and we saw no evidence of agonistic encounters between translocated and resident elephants. Therefore, consistent with non-territoriality of elephants [39], translocated individuals are also unlikely to have left the parks due to antagonism by resident elephants.

### Individual Variation

Individual elephants responded variably to translocation by homing back, wandering or settling, the type of response being unrelated to translocation distance. In an assessment of elephant re-introductions in South Africa, no factors including distance explained translocation failure [45]. We found 3 of 5 mature-adults and none of 7 young-adults displayed successful homing movements, suggesting a tendency of successful homing by older individuals (Table 3). Many species show individually variable responses to translocation with some returning to the capture site and others settling at the release location [10], [11]. In some species the probability of returning home is inversely related to distance translocated (wolves [11], bears [49], foxes [12], Gila monsters [20]) and in some, those that return are more likely to be adults (cougars [13], wolves [11]). Sex bias with males more likely to leave has been observed in cougars [13] and black bears [3]. Individual response to translocation may also be related to environmental factors such as relative resource availability of capture and settling/release locations, physiographic and anthropogenic barriers; behavioral factors such as social status, and covert aggression of conspecifics; and innate factors such as physiological and psychological states of individuals. However, such aspects are difficult to test empirically.

### Extent of Ranging

Use-areas of translocated elephants were significantly larger than home ranges of resident elephants. On the three instances translocated elephants returned home, their use-areas between release and return were greater than their post-return home ranges. Wider ranging upon translocation has been documented in many species [3] including cougars [13], black bears [57], snakes [21] and crocodiles [22]. In addition to attempted homing, animals released in occupied habitats may show increased ranging due to competition with residents and exploration. Given the apparent resource abundance and the absence of overt aggression from conspecifics, the increased post-translocation ranging observed in our study maybe primarily explained by attempted homing and secondarily by exploration.

### Relation to HEC

Practically all translocated elephants were involved in HEC post-release. They ranged widely with ‘Homers’ and ‘wanderers’ venturing outside normal elephant range, some even entering highly populated cities. Thus, problem-elephant translocation resulted in wider propagation of HEC.

Translocated elephants roamed in environments alien to them, in ignorance of the lay of the land. This increased the likelihood of unanticipated encounters and conflict with humans. The 12 resident males did not cause any human deaths. This finding is consistent with the annual elephant induced human mortality rate in Sri Lanka (including that by about 14 elephants translocated annually) of 0.04 humans/adult male or 0.01 humans/elephant [28]. In contrast, human mortality caused by the 12 translocated elephants monitored was an order of magnitude higher at 0.42 humans/elephant (Fisher's exact test, p<0.0001). Therefore problem-elephant translocation intensified HEC.

Most translocated elephants resumed raiding after release (Table 3). Elephants in shared landscapes are preferential, rather than obligate raiders [58]. Therefore, raiders are likely to be compulsive and continue to raid irrespective of changed circumstances. Post-release assessments of behaviors characterizing problem-animals have been few, but most have found lack of reform [3], [5]. A study of house-denning raccoons found the majority to persist with the behavior after removal [9]. Of four tigers translocated because of livestock predation, two immediately moved to human dominated habitats [15]. Three of four translocated stock-raiding leopards resumed raiding [59]. A survey of leopard translocations found a positive correlation between translocations and conflict [14]. Translocation was found to be largely unsuccessful at keeping problem wolves out of livestock production areas [60]. Our findings are consistent with these observations and suggest that ‘successful’ problem-animal translocation most likely translocates not only the animal but also the problem.

Galli and Wasaba did not raid post-release. Translocation is the culmination of a train of events, usually instigated by a major incident like human death or house breaking by elephants. Capture occurs days to weeks after the incident. Elephants in Sri Lanka have home ranges of 41–643 km^2^ (Table 2) [39]. Consequently, by the time of capture the elephant responsible may no longer be in the vicinity. Additionally, most HEC incidents occur at night and even if witnessed, the perpetrator cannot be identified with certainty. Thus, Galli and Wasaba may not have been problem-elephants but victims of ‘mistaken-identity’.

### Release Type

Reviews of avian and mammal translocations have generally found a greater number of ‘successful’ translocations with hard-release [2], [61]. While IUCN guidelines for African elephant translocation recommend soft-release [17], some re-introduced African elephants so translocated still left the release area [45]. Effect of release type has mostly been assessed in re-introductions, where settling in the release area denotes success. All the elephants in our study were hard-released and some settled near release areas but reverted to raiding. It is unlikely that release type would have much bearing on the outcome in problem-animal translocation, where eliminating the problem is the primary objective [6] and its translocation signifies failure.

Soft-release is also advocated in African elephant translocation for ‘educating’ elephants to respect electric fences during acclimatization [17]. All elephants who encountered electric fences in our study broke through them. In Galli’s case, breakout occurred only after months of fence patrolling, suggesting sustained effort to overcome fences rather than a lack of respect for them. Therefore, the effect of release type on fence breaking is debatable. However, the adequacy of the fences that translocated elephants were confronted with could be a confounding factor.

### Survival of Translocated Elephants

The 12 resident males tracked had a death rate of 0.10 per tracked-elephant-year. This is consistent with the annual mortality rate of adult male elephants in Sri Lanka of around 7–8% [28]. All 12 translocated elephants survived to adulthood in their original home ranges. However, five of them died within 8 months of release (Table 3), amounting to 42% mortality or 0.44 deaths per tracked-elephant-year. Additionally, translocation carries a mortality rate of approximately 6% during capture and transport [28]. Therefore, although translocation aimed to safeguard ‘problem-elephants’, in reality it greatly reduced their survival.

Increased mortality of translocated individuals has been observed in raccoons [62], cougars [13], wolves [11], elephants [35] and snakes [21]. Similar survivability to resident populations has been reported in muskoxen [63]. Some studies found increased mortality in black bears [3] while others did not [49], [57]. Higher mortality of translocated animals may be related to their wider ranging in unfamiliar environments. Additionally, ‘problem-animals’ are individuals with a greater predilection for conflict with people and the probability of encounters hence conflict is increased by translocation. Therefore, as seen in our study, mortality is likely to be much higher in translocated problem-animals.

### Ethical Implications

Translocation caused elephants to behave abnormally, increased their mortality, and presumably subjected individuals to extreme stress. Elephants are a highly social species with a network of relationships even amongst males [64]. Translocation disrupts such relationships at both capture and release locations. Elephants are also an intelligent and long-lived species. Consequently, profound negative experiences may have extensive and long-term psycho-physiological effects on their brains and behavior [65]. Therefore, from an elephant welfare point of view, translocation is not an acceptable management tool.

### Conclusion

We conclude that problem-elephant translocation causes intensification and broader propagation of HEC and increased elephant mortality, hence defeats both HEC mitigation and elephant conservation goals. The driver of translocation is public and political pressure. Capturing and translocating an elephant from the vicinity of major HEC incidents may defuse tension hence be of relevance in particular contexts. However we found that even if the original problem is solved by translocation, the same or more likely worse is created at another location.

Based on our results we advocate phasing out problem-elephant translocation, for which public awareness is key. In the interim, translocations should only be undertaken with monitoring through GPS-telemetry, and contingency plans to address unintended outcomes. Problem-elephant translocation without either, amounts to reckless disregard for the safety and welfare of people and elephants. In the long term, attention needs to be shifted towards preventing the genesis of ‘problem-elephants’. Such a strategy requires eliminating elephant management and crop protection methods that promote elephant aggression and increase HEC, and implementing land-use plans that minimize crop raiding.

## References

[1] IUCN (1998) Guidelines for Re-introductions. Prepared by the IUCN/SSC Re-introduction Specialist Group. Gland, Switzerland and Cambridge, UK: IUCN.

[2] Wolf CM, Griffith B, Reed C, Temple SA (1996) Avian and Mammalian Translocations: Update and Reanalysis of 1987 Survey Data. Conserv Biol 10: 1142–1154.

[3] Linnell JDC, Aanes R, Swenson JE, Odden J, Smith ME (1997) Translocation of carnivores as a method for managing problem animals: a review. Biodiv Conserv 6: 1245–1257.

[4] Craven S, Barnes T, Kania G (1998) Toward a professional position on the translocation of problem wildlife. Wildl Soc Bull 26: 171–177.

[5] Loveridge AJ, Wang SW, Frank LG, Seidensticker J (2010) People and wild felids: conservation of cats and management of conflicts. In: Macdonald DW, Loveridge AJ, editors. The Biology and Conservation of Wild Felids. New York: Oxford University Press. 161–195.

[6] Massei G, Quy RJ, Gurney J, Cowan DP (2010) Can translocations be used to mitigate human–wildlife conflicts? Wildl Res 37: 428–439.

[7] Treves A, Karanth KU (2003) Human-carnivore conflict and perspectives on carnivore management worldwide. Conserv Biol 17: 1491–1499.

[8] Loredo-Prendeville I, Vuren DV, Kuenzi AJ, Morrison ML (1994) California ground squirrels at Concord naval weapons station: alternatives for control and the ecological consequences. In: Halverson WS, Crabb AC, editors. Proceedings of the Sixteenth Vertebrate Pest Conference. Davis, CA: University of California.

[9] O’Donnell MA, DeNicola AJ (2006) Den site selection of lactating female raccoons following removal and exclusion from suburban residences. Wildl Soc Bull 34: 366–370.

[10] Rogers LL (1988) Homing tendencies of large mammals: a review. In: Nielsen L, Brown R, editors. Translocation of Wild Animals. Milwaukee, WI: Wisconsin Humane Society. 76–92.

[11] Bradley EH, Pletscher DH, Bangs EE, Kunkel KE, Smith DW, et al (2005) Evaluating wolf translocation as a nonlethal method to reduce livestock conflicts in the northwestern United States. Conserv Biol 19: 1498–1508.

[12] Lenain DM, Warrington S (2001) Is translocation an effective tool to remove predatory foxes from a desert protected area? J Arid Environ 48: 205–209.

[13] Ruth TK, Logan KA, Sweanor LL, Homocker MG, Temple LJ (1998) Evaluating cougar translocations in New Mexico. J Wildl Manag 62: 1264–1275.

[14] Athreya V, Odden M, Linnell JDC, Karanth KU (2010) Translocation as a tool for mitigating conflict with leopards in human-dominated landscapes of India. Conserv Biol 25: 133–141. 21054526 10.1111/j.1523-1739.2010.01599.x

[15] Goodrich JM, Miquelle DG (2005) Translocation of problem Amur tigers *Panthera tigris altaica* to alleviate tiger-human conflicts. Oryx 39: 1–4.

[16] Fernando P (1997) Keeping jumbo afloat. Sri Lanka Nature 1: 4–12.

[17] Dublin HT, Niskanen LS (editors) (2003) IUCN/SSC AfESG Guidelines for the in situ Translocation of the African Elephant for Conservation Purposes. Gland, Switzerland: IUCN.

[18] Holevinski RB, Malecki RA, Curtis PD (2006) Can hunting of translocated nuisance Canada geese reduce local conflicts? Wildlife Soc Bull 34: 845–849.

[19] Boshoff AF, Vernon CJ (1988) The translocation and homing ability of problem eagles. S Afr J Wildlife Res 18: 38–40.

[20] Sullivan BK, Kwiatkowski MA, Schuett GW (2004) Translocation of urban Gila monsters: a problematic conservation tool. Biol Conserv 117: 235–242.

[21] Butler H, Malone B, Clemann N (2005) The effects of translocation on the spatial ecology of tiger snakes (*Notechis scutatus*) in a suburban landscape. Wildlife Res 32: 165–171.

[22] Read MA, Grigg GC, Irvin SR, Shanahan D, Franklin CE (2007) Satellite tracking reveals long distance coastal travel and homing by translocated estuarine crocodiles. *Crocodylus porosus* . PloS ONE 9: 1–5. 10.1371/journal.pone.0000949 PMC1978533 17895990

[23] IUCN (2011) IUCN Red List of Threatened Species. Version 2011.2. Available: http://www.iucnredlist.org. Accessed 2012 Nov 10. (Current version: 2012.2.).

[24] Santiapillai C, Jackson P (1990) The Asian Elephant: An Action Plan for its Conservation. Gland, Switzerland: IUCN/SSC Asian Elephant Specialist Group.

[25] Fernando P, Pastorini J (2011) Range-wide status of Asian elephants. Gajah 35: 15–20.

[26] Fernando P, Leimgruber P (2011) Asian elephants and dry forests. In: McShea WJ, Davies SJ, Phumpakphan N, Pattanavibool A, editors. The Ecology and Conservation of Seasonally Dry Forests in Asia. Washington, DC: Smithsonian Institution Scholarly Press. 151–163.

[27] Leimgruber P, Gagnon JB, Wemmer C, Kelly DS, Songer MA, et al (2003) Fragmentation of Asia’s remaining wildlands: implications for Asian elephant conservation. Anim Conserv 6: 347–359.

[28] Fernando P, Jayewardene J, Prasad T, Hendavitharana W, Pastorini J (2011) Current status of Asian elephants in Sri Lanka. Gajah 35: 93–103.

[29] Moss CJ, Poole JH (1983) Relationships and social structure of African elephants. In: Hinde RA, editor. Primate Social Relations: An Integrated Approach. Oxford: Blackwell Scientific Publications. 315–325.

[30] Sukumar R (1989) The Asian Elephant: Ecology and Management. Cambridge: Cambridge University Press.

[31] Fernando P, Lande R (2000) Molecular genetic and behavioral analyses of social organization in the Asian elephant. Behav Ecol Sociobiol 48: 84–91.

[32] Sukumar R (1991) The management of large mammals in relation to male strategies and conflict with people. Biol Conserv 55: 93–102.

[33] Fernando P (2011) Managing ‘problem elephants’. Loris 25(6): 32–36.

[34] Slotow R, Whyte I, Hofmeyr M, Kerley GHI, Conway T, et al.. (2008) Lethal management of elephants. In: Scholes RJ, Mennell KG, editors. Elephant Management: A Scientific Assessment of South Africa. Johannesburg: Witwatersrand University Press.

[35] Pinter-Wollman N (2009) Spatial behaviour in translocated African elephants (*Loxodonta africana*) in a novel environment: using behaviour to inform conservation. Behaviour 146: 1171–1192.

[36] Saaban S, Othman NB, Yasak MNB, Burhanuddin MN, Zafir A, et al (2011) Current status of Asian elephants in Peninsular Malaysia. Gajah 35: 67–75.

[37] Stuewe M, Abdul JB, Nor BM, Wemmer CM (1998) Tracking the movements of translocated elephants in Malaysia using satellite telemetry. Oryx 32: 68–74.

[38] Douglas-Hamilton I, Krink T, Vollrath F (2005) Movements and corridors of African elephants in relation to protected areas. Naturwissenschaften 92: 158–163. 15770465 10.1007/s00114-004-0606-9

[39] Fernando P, Wickramanayake ED, Janaka HK, Jayasinghe LKA, Gunawardene M, et al (2008) Ranging behavior of the Asian elephant in Sri Lanka. Mammal Biol 73: 2–13.

[40] Roy M, Choudhury SP, Kamalakanth P, Dutta C, Kundu S, et al (2010) Translocation of a wild elephant from southern West Bengal to northern West Bengal–An approach to reduce elephant-human conflict. Gajah 33: 8–11.

[41] Lin L, Feng L, Pan W, Guo X, Zhao J, et al (2008) Habitat selection and the change in distribution of Asian elephants in Mengyang Protected Area, Yunnan, China. Acta Theriol 53: 365–374.

[42] Slotov R, Van Dyk G (2004) Ranging of older male elephants introduced to an existing small population without older males: Pilanesberg National Park. Koedoe 47: 91–104.

[43] Banks J (1979) The translocation of the Deduru Oya herd. What was left of it. Loris 15: 113–115.

[44] Lahiri-Choudhury DK (1993) Problems of wild elephant translocation. Oryx 27: 53–55.

[45] Garai ME, Carr RD (2001) Unsuccessful introductions of adult elephant bulls to confined areas in South Africa. Pachyderm 31: 52–57.

[46] Arivazhagan C, Sukumar R (2008) Constructing age structures of Asian elephant populations: A comparison of two field methods of age estimation. Gajah 29: 11–16.

[47] Varma S, Baskaran N, Sukumar R (2012) Field Key for Elephant Population Estimation and Age and Sex Classification. Bangalore: Asian Nature Conservation Foundation, Innovation Centre, Indian Institute of Science.

[48] Miller SD, Ballard WB (1982) Homing of transplanted Alaskan brown bears, J Wildl Manage. 46: 869–876.

[49] Rogers LL (1986) Effects of translocation distance on frequency of return by adult black bears. Wildl Soc Bull 14: 76–80.

[50] Eberhardt LL, Pickens HC (1979) Homing tendencies in mule deer. Southwest Nat 24: 705–706.

[51] Oliver GW, Morris PA, Thorson PH, Le Boeuf BJ (1998) Homing behavior of juvenile northern elephant seals. Marine Mammal Sci 14: 245–256.

[52] Phillips JB, Adler K, Borland SC (1995) True navigation by an amphibian. Anim Behav 50: 855–858.

[53] Burt WH (1943) Territoriality and home range concepts as applied to mammals. J Mammal 24: 346–352.

[54] Sandell M (1989) The mating tactics and spacing patterns of solitary carnivores. In: Gittleman JL, editor. Carnivore Behaviour, Ecology and Evolution. Ithaca: Cornell University Press. 164–182.

[55] Shier DM, Swaisgood RR (2012) Fitness costs of neighborhood disruption in translocations of a solitary mammal. Conserv Biol 26: 116–123. 21978094 10.1111/j.1523-1739.2011.01748.x

[56] Baskaran N, Desai AA (1996) Ranging behavior of the Asian elephant (*Elephas maximus*) in the Nilgiri biosphere reserve, South India. Gajah 15: 41–57.

[57] Benson JF, Chamberlain MJ (2007) Space use, survival, movements, and reproduction of reintroduced Louisiana black bears. J Wildl Manag 71: 2393–2403.

[58] Ekanayaka SKK, Campos-Arceiz A, Rupasinghe M, Pastorini J, Fernando P (2011) Patterns of crop raiding by Asian elephants in a human-dominated landscape in Southeastern Sri Lanka. Gajah 34: 20–25.

[59] Weilenmann M, Gusset M, Mills DR, Gabanapelo T, Schiess-Meier M (2010) Is translocation of stock-raiding leopards into a protected area with resident conspecifics an effective management tool? Wildl Res 37: 702–707.

[60] Fritts SH, Paul WJ, Mech LD (1984) Movements of translocated wolves in Minnesota. J Wildl Manag 48: 709–721.

[61] Short J (2009) Australian Animal Welfare Strategy–The Characteristics and Success of Vertebrate Translocations within Australia. Canberra, Australia: Australian Government Department of Agriculture, Fisheries and Forestry.

[62] Allen TJ (1986) Evaluation of movements, harvest rate, vulnerability and survival of translocated raccoons in southern West Virginia. Trans Northeast Sect Wildlife Soc 43: 64.

[63] Le Henaff D, Crete M (1989) Introduction of muskoxen in northern Quebec: the demographic explosion of a colonizing herbivore. Can J Zool 67: 1102–1105.

[64] Chiyo PI, Archie EA, Hollister-Smith JA, Lee PC, Moss CJ, Alberts SC (2011) Association patterns of African elephants in all-male groups: the role of age and genetic relatedness. Anim Behav 81: 1093–1099.

[65] Bradshaw GA, Schore AN, Brown JL, Poole JH, Moss CJ (2005) Elephant breakdown. Nature 433: 807. 15729320 10.1038/433807a

